# Use of an Extract of *Annona muricata* Linn to Prevent High-Fat Diet Induced Metabolic Disorders in C57BL/6 Mice †

**DOI:** 10.3390/nu11071509

**Published:** 2019-07-02

**Authors:** Sandramara Sasso, Priscilla Cristovam Sampaio e Souza, Lidiani Figueiredo Santana, Claudia Andréa Lima Cardoso, Flávio Macedo Alves, Luciane Candeloro Portugal, Bernardo Bacelar de Faria, Anderson Fernandes da Silva, Ana Rita Coimbra Motta-Castro, Luana Silva Soares, Larissa Melo Bandeira, Rita de Cássia Avellaneda Guimarães, Karine de Cássia Freitas

**Affiliations:** 1 Posgraduate Program in Health and Development in the Midwest Region, Medical School, Federal University of Mato Grosso do Sul, Campo Grande, 79070-900 Mato Grosso do Sul, Brazil; 2 Faculty of Pharmaceutical Sciences, Food and Nutrition, Federal University of Mato Grosso do Sul, Campo Grande, 79070-900 Mato Grosso do Sul, Brazil; 3 Course of Chemistry, State University of Mato Grosso do Sul, Dourados, 79070-900 Mato Grosso do Sul, Brazil; 4 Institute of Biosciences, Federal University of Mato Grosso do Sul, Campo Grande, 79070-900 Mato Grosso do Sul, Brazil; 5 Medicina Diagnóstica Laboratory-Scapulatempo, Campo Grande, 79002-170 Mato Grosso do Sul, Brazil; 6 Laboratory of Clinical Immunology, Faculty of Pharmaceutical Sciences, Food and Nutrition, Federal University of Mato Grosso do Sul, Campo Grande, 79070-900 Mato Grosso do Sul, Brazil; 7 Oswaldo Cruz Foundation, Campo Grande, 79074-460 Mato Grosso do Sul, Brazil

**Keywords:** graviola, weight loss, obesity

## Abstract

*Annona muricata* Linn, commonly known as graviola, is one of the most popular plants used in Brazil for weight loss. The aim of this study is to evaluate the therapeutic effects of three different doses (50 mg/kg, 100 mg/kg, and 150 mg/kg) of aqueous graviola leaf extract (AGE) supplemented by oral gavage, on obese C57BL/6 mice. Food intake, body weight, an oral glucose tolerance test (OGTT), an insulin sensitivity test, quantification of adipose tissue cytokines, weight of fat pads, and serum biochemical and histological analyses of the liver, pancreas, and epididymal adipose tissue were measured. AGE had an anti-inflammatory effect by increasing IL-10 at doses of 50 and 100 mg/kg. Regarding the cholesterol profile, there was a significant decrease in LDL-cholesterol levels in the AGE 150 group, and VLDL-cholesterol and triglycerides in the AGE 100 and 150 groups. There was an increase in HDL cholesterol in the AGE 150 group. The extract was able to reduce the adipocyte area of the epididymal adipose tissue in the AGE 100 and 150 groups. According to the histological analysis of the liver and pancreas, no significant difference was found among the groups. There were no significant effects of AGE on OGTT and serum fasting glucose concentration. However, the extract was effective in improving glucose tolerance in the AGE 150 group.

## 1. Introduction

Obesity is a worldwide public health problem. It increases the risk of metabolic diseases such as hypercholesterolemia, hypertriglyceridemia, insulin resistance, heart disease, type 2 diabetes, atherosclerosis, and cancer [1,2].

The etiology of obesity is complex and multifactorial. Obesity results from the interaction of genetic/epigenetic, environmental, emotional, lifestyle factors and that technically obesity results from a positive energy balance: More energy intake than energy expenditure [3]. Although genetic factors are determinant in the development of obesity, metabolic factors, an unhealthy diet, and a sedentary lifestyle provide conditions for the development of this disorder [4].

Obesity is an increased deposition of white adipose tissue and phenotypic changes in this tissue. It is associated with metabolic changes such as increased production of pro-inflammatory mediators. This leads to organs dysfunction and chronic low-grade inflammation with high levels of proinflammatory cytokines, such as interleukin-6 (IL-6) and tumor necrosis factor-α (TNF-α), and chemokines, such as monocyte chemotactic protein 1 (MCP1), which in turn promotes migration of macrophages into the adipose tissue and increases the release of cytokines. In parallel, low levels of interleukin 10 (IL-10) are observed in obese individuals, and worsens their metabolic profile, since IL-10 inhibits the synthesis of pro-inflammatory cytokines [2,5,6,7,8].

To control such abnormalities, several methods have been suggested to regulate obesity and weight gain, including agents that could inhibit fat absorption, control biochemical parameters, such as, serum glucose, serum triglyceride, total cholesterol, high-density lipoprotein (HDL), low-density lipoprotein (LDL), and very low-density lipoprotein (VLDL) levels, reduce systemic inflammation, and induce weight loss [9]. Medicinal plants, popularly indicated for the treatment of obesity, have been used in many countries to control weight gain and obesity [10].

For these reasons, anti-obesity agents, including infusions and extracts, are widely used for weight control in obese individuals, in addition to reducing biochemical parameters [11].

Tropical countries have a wide variety of flora and a high number of food and medicinal plants. There is information available on the potential functional properties of several of these plants [12]. The investigation of such properties may be of interest for both the pharmaceutical and the food industry [13].

Among the species of pharmaceutical interest, the leaf of *Annona muricata* Linn (Annonaceae), commonly known as soursop or graviola, is used routinely for weight control. It is used in traditional medicine as an antihypertensive, vasodilator, antidiabetic, and hypolipidemic agent due to the presence of several bioactive compounds, such as acetogenins, flavonoids, tannins, alkaloids, coumarins, and terpenoids [14,15]. Therefore, considering the popular use of tea from graviola leaves to prevent obesity and its complications, it is important to verify whether treatment using an aqueous extract of *Annona muricata* Linn could also be beneficial for the treatment of obesity. Thus, the objective of this study is to verify the effects of three different doses of aqueous extract of *Annona muricata* on obese C57BL/6 mice induced by a high-fat diet.

## 2. Materials and Methods

### 2.1. Extraction of Plant Material

Leaves of *Annona muricata* Linn were collected in June 2015 from an adult specimen that produces flowers and fruits, in the municipality of Campo Grande, Mato Grosso do Sul state, Brazil. The tree was properly identified. The geographical coordinates defined by manual GPS were 22°29′42.6″ S and 054°37′1.6″ W. A voucher specimen (number 53,928) was deposited at the Herbarium CGMS of the Federal University of Mato Grosso do Sul, Brazil. The extract of leaves of *Annona muricata* Linn was prepared by immersing 1 kg of leaf powder into 3 L of distilled water for 48 h, then lyophilizing this until a dry powder was obtained. Then, the extract was stored at room temperature and protected from light until use [14].

### 2.2. Quantification of Total Phenols and Flavonoids

The total phenols of aqueous graviola leaf extract (AGE) were determined by the Folin-Ciocalteu reagent method [16]. Samples and a standard curve of gallic acid were read at 760 nm. The result was expressed as mg of gallic acid per g of extract. For the quantification of the flavonoids, the colorimetric method of aluminum chloride was used [17]. The absorbances were read at 415 nm with a UV-Vis spectrophotometer. To calculate the concentration of flavonoids, an analytical curve was prepared using quercetin as standard. The results are expressed as mg quercetin per g of extract.

### 2.3. Quantification of Condensed Tannins

The extract was dissolved in water at a concentration of 50 μg·mL^−1^ using the valinine reaction [18]. The absorbance reading was performed using a spectrophotometer at 510 nm. The quantification was performed using an external calibration curve with catechin as standard. The results are expressed as mg equivalent of catechin per g of extract.

### 2.4. Assay of Antioxidant Activity Using the 2,2-Diphenyl-1-Picrylhydrazyl Free Radical (DPPH)

The sequestering capacity was measured using DPPH solution. The absorbances were read at 517 nm with a spectrophotometer. The percentage of DPPH radical sequestration inhibition was calculated according to the equation:

Percent inhibition activity (%) = [(A0 − A1)/A0] 100(1)

where *A*_0_ is the absorbance of the control, and *A*_1_ is the absorbance in the presence of the compound. Subsequently, the mean inhibitory concentration (IC 50) was calculated. It represents the concentration of the sample required to capture 50% of the DPPH [19].

### 2.5. Isolation and Identification of Compounds

The extract was fractionated by XAD-2 (Supelco, Bellefonte, PA, USA) on column chromatography (30 cm × 3 cm). The extract (3.16 g) was eluted with 0.5 L of water, followed by 0.5 L of methanol, and again eluted with 0.2 L of ethyl acetate. An aliquot of 0.89 g of the methanolic fraction was dissolved into 50 mL of methanol and fractionated by chromatography using a Sephadex LH-20 (Amersham Pharmacia Biotech, Uppsala, Sweden) on column chromatography (70 cm × 3 cm) at a rate of 0.2 mL·min^−1^. Twenty-five fractions of 2 mL were collected. The fractions were combined according to their chemical behavior on thin layer chromatography (silica gel plates) using ethyl acetate:n-propanol:water (123:7:70 *v*/*v*/*v*) as the eluent. The fractions 2–4, 6–8 and 11–14 were purified using polyvinylpolypyrrolidone (Sigma, St. Louis, MO, USA) on column chromatography (10 cm × 2 cm) by eluting them with methanol. The result is the identification of compounds. An aliquot of 0.54 g of the ethyl acetate fraction was dissolved into 10 mL of methanol and fractionated by chromatography using a Sephadex LH-20 (Amersham Pharmacia Biotech, Uppsala, Sweden) on column chromatography (80 cm × 2 cm) by eluting it with methanol at a rate of 0.3 mL·min^−1^. Twenty-eight fractions of 5 mL were collected. The fractions were combined according to their chemical behavior on thin layer chromatography (silica gel plates) using ethyl acetate:methanol (60:40 *v*/*v*) as the eluent. The fractions 10–13, 18–19 and 22–25 resulted in the isolation of the other compounds. The identification of the compounds was carried out using ^1^H and ^13^C nuclear resonance (Advance 300 MHz, Brucher, Ettlingen, Germany) and mass spectrometry (Shimadzu Corp. Shimadzu, Kyoto). Their chemical structures were confirmed by comparison with literature data [20,21,22].

### 2.6. Ethics Statement

All animal experiments were submitted and approved by the Ethics Committee on Animal Use, Federal University of Mato Grosso do Sul (Protocol n^o^. 682/2015).

### 2.7. Acute Oral Toxicity

The acute toxicity test of the AGE was performed in female Wistar rats (*Rattus norvegicus*) based on the OECD Guidelines 425 (Organization for Economic Co-operation and Development) [23]. For the test, the animals were divided into two groups (*n* = 5): A control group that received saline solution, and the treatment group that received the aqueous extract of *Annona muricata* Linn orally (gavage) at a dose of 2000 mg/kg. After treatment, the animals were observed at 30 min, 1 h, 2 h, 3 h, 4 h, 6 h, 12 h, 24 h, and then daily for 14 days.

At the same time, the hippocratic screening test was carried out to quantify the effects of abnormal morphological and behavioral signs of toxicity. Furthermore, changes in body weight, water and food intake, as well as excreta production, were also evaluated [24].

At the end of 14 days, the animals were euthanized (ketamine and xylazine). The organs (heart, lung, liver, spleen, pancreas, and kidneys) were removed, weighed, and analyzed macroscopically to investigate possible changes [25].

### 2.8. Animals and Experimental Design

C57BL/6 adult male mice (*n* = 55, 12 weeks of age) were divided into two groups based on body weight, as follows: SHAM group (*n* = 11), treated with standard diet AIN-93M [26], and HFD group (*n* = 44), treated with a hyperlipidic diet. After 12 weeks, the animals of the HFD group were divided into four homogenous groups according to weight and value of fasting blood glucose and concomitantly supplemented (oral gavage) with aqueous graviola leaf extract in different doses: HFD SALINE group (HFD + saline), AGE 50 mg/kg group (HFD + aqueous graviola leaf extract of 50 mg/kg) (*n* = 11), AGE 100 mg/kg group (HFD + aqueous graviola leaf extract of 100 mg/kg) (*n* = 11), and AGE 150 mg/kg group (HFD + aqueous graviola leaf extract of 150 mg/kg) (*n* = 11). The SHAM group also received saline solution at this stage of the study. Each group had ad libitum access to water and food during the experimental period. The composition of the experimental diets is show in the Table 1 below.

The mice were anesthetized (Ketamine and xylazine, 75 and 10 mg/kg, respectively), and euthanized by cardiac puncture when they reached 35 weeks of age. The blood and the organs were collected for subsequent analyses.

### 2.9. Body Weight and Diet Intake

The mice were weighed weekly to observe weight changes until the end of the study. Food intake was measured three times per week.

The energy intake was calculated by multiplying the amount of diet ingested (g/day/animal) by the energy density of each diet, expressed in kcal/day per animal. In addition, the calculation of the feed efficiency index (FEI) was performed using the following equation:

(2) $$\mathrm{Free}\;\mathrm{efficiency}\;\mathrm{index}\;=\frac{\left(FW-IW\right)}{TF}$$

where *FW* is the final body weight in grams, *IW* is the initial body weight in grams, and *TF* is the total amount of food ingested in grams [27].

### 2.10. Biochemical Analysis

Serum glucose, serum triglyceride, total cholesterol, high-density lipoprotein (HDL), low-density lipoprotein (LDL), and very low-density lipoprotein (VLDL) levels were analyzed by the enzymatic colorimetric test, according to the manufacturer’s instructions (Labtest^®^, Lagoa Santa, Minas Gerais, Brazil). The atherogenic index was determined by the ratio between total cholesterol and HDL cholesterol [14].

### 2.11. Oral Glucose Tolerance Test

The oral glucose tolerance test (OGTT) was performed one day prior to initiating treatment with the AGE or saline solution, and three days prior to the euthanasia of animals after six hours of fasting. Fasting glucose was verified via flow rate (time 0) using a G-Tech^®^ glucometer (G-TECH Free, Infopia Co., Ltd. South Korea). Then, the animals received a D-glucose solution (Sigma Aldrich, Duque de Caxias, Rio de Janeiro, Brazil), at 2 g/kg of body weight, by gavage. A blood glucose reading was performed 15, 30, 60 and 120 min after glucose application. The area under the curve (AUC) was calculated for each mouse, and the mean was calculated for each experimental group [28].

### 2.12. Insulin Sensitivity Test

The insulin sensitivity test was performed five days before euthanasia. Glycemia was verified with the animals in a fed state (time 0). Then, 0.75 units of insulin (NovoRapid^®^, 100 U/mL, Novo Nordisk, Bagsvaerd, Denmark) per kg of animal weight was injected intraperitoneally. The blood glucose reading was performed at 15, 30 and 60 min using a G-Tech^®^ glucometer (G-TECH Free, Korea). The area under the curve (AUC) was calculated for each animal, and the mean was calculated for each experimental group [28].

### 2.13. Quantification of Cytokines in the Adipose Tissue

Epididymal adipose tissue was collected, weighed (100 mg) and stored at –80 °C. For protein extraction, the epididymal adipose tissue was thawed on ice and homogenized in 1 mL of RIPA (RIPA Lysis Buffer, 10×, Cat. n^o^. 20–188, MERCK, Darmstadt, Germany). A cocktail of protease inhibitors was added (Protease Inhibitor Cocktail Set Calbiochem, Cat. n^o^. 539131, MERCK, Darmstadt, Germany).

The supernatant was collected after centrifugation at 4 °C and stored again at –80 °C until cytokine analysis, according to the recommendations of the manufacturer (MILLIPLEX MAP/Mouse Cytokine/Chemokine and Adipocyte Magnetic Bead panel) (Millipore, Billerica, MA, USA). The concentrations of the following cytokines were analyzed: IL-10, IL-6, MCP-1, and TNF-α using the MCYTOMAG-70K kit, and adiponectin using the MADCYMAG-72K kit. The concentration of the cytokines IL-10, IL-6, MCP-1, and TNF-α in the adipose tissue was expressed as cytokine picograms in relation to protein content (mg of protein). For adiponectin, the values were expressed as nanograms of cytokines in relation to protein content (mg of protein). Protein quantification was based on the bicinchoninic acid assay (BCA) following the manufacturer’s recommendations (BCA Protein Assay kit) (MERCK, Darmstadt, Germany) [29,30].

### 2.14. Assessment of Body Fat and Liver Weight

After euthanasia, the liver and fat pads of white adipose tissue (omental, epididymal, perirenal, retroperitoneal, and mesenteric) were dissected and weighed. The adiposity index was calculated as the total sum of visceral white adipose tissue (g) divided by the final body weight of the animal x 100 and expressed as percentage of adiposity [31].

### 2.15. Histopathological Analysis

Samples of the liver, pancreas, and epididymal adipose tissue were fixed with 10% formalin solution. After fixation, the specimens were dehydrated, embedded in paraffin, cut in a microtome to a thickness of 5 mm each, and stained with hematoxylin-eosin. An expert pathologist performed the histological analysis of the liver and pancreas. For the analysis of treatment effects on the hepatocytes, a scoring system was used [32]. In the evaluation of the architecture of the pancreas, there were changes in the Islets of Langerhans and pancreatic acini, and inflammation was observed [33,34]. For the analysis of the adipocyte area of the epididymal adipose tissue, the images were initially taken using a LEICA DFC 495 digital camera system (Leica Microsystems, Wetzlar, Germany) integrated into a LEICA DM 5500B microscope (Leica Microsystems, Wetzlar, Germany), with a magnification of 20X. The images were analyzed using the LEICA Application Suite software, version 4.0 (Leica Microsystems, Wetzlar, Germany), and the mean area of 100 adipocytes per sample was determined [35].

### 2.16. Statistical Analyses

The results were expressed as mean ± MSE (mean standard error). For multiple comparisons of parametric results, an ANOVA followed by a Tukey post-test were performed. The Student t-test was performed for comparison between two groups. The chi-square test was used to evaluate associations in histological analyses. A significance level of *p* < 0.05 was adopted. Statistical analysis was performed using the software Jandel Sigma Stat, version 3.5 (Systat software, Incs., San Jose, CA, USA), and Sigma Plot, version 12.5 (Systat Software Inc., San Jose, CA, USA).

## 3. Results

### 3.1. Chemical Composition

The content of phenols, flavonoids, and tannins in AGE was 156.37 ± 1.2 mg/g, 92.07 ± 1.8 mg/g and 42.99 ± 0.6 mg/g, respectively. The antioxidant activity of IC_50_ was 12 ± 0.1 μg·mL^−1^. In addition, six compounds were isolated and identified in the extract: kaempferol-3-*O*-a-l-rhamnopyranoside, quercetin 3-*O*-rutinoside, kaempferol 3-*O*-rutinoside, luteolin, quercetin, and sitosterol-3-*O*-β-d-glucopyranoside.

### 3.2. Acute Oral Toxicity

The results showed no signs of systemic toxicity. There are no changes in body weight, water consumption, food intake, and excretion of urine and feces. In addition, no changes in the Hippocratic screening test were observed, such as motor and/or sensory and neurological changes, as no animals died. The weight of the liver, spleen, pancreas, lungs, heart, and kidneys did not show significant differences among groups. Macroscopic changes in the organs of the animals were not visualized (Supplementary Material Figure S1).

### 3.3. Effects of AGE on Body Weight and Food Intake

At the beginning of the experiment, the animals in the HFD group did not present significant differences in body weight when compared to animals in the SHAM group (*p* = 0.971) (Table 2).

**Figure 1:**
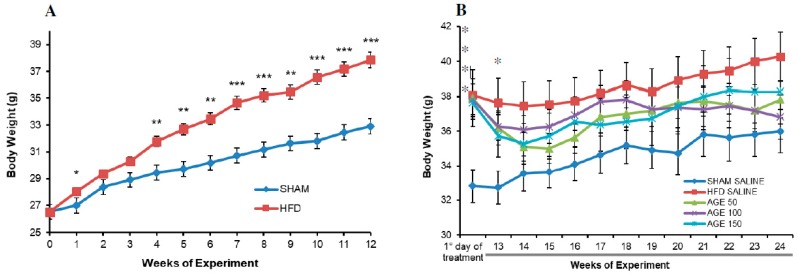
Effects of a hyperlipidic diet and AGE on weight gain. (**A**) Body weight of animals fed on the standard diet (SHAM) and on the hyperlipidic diet (HFD) during obesity induction for 12 consecutive weeks (0: initial weight). (**B**) Weight of control animals (SHAM SALINE: standard diet + saline solution. HDF SALINE: hyperlipidic diet + saline solution) and of animals treated with aqueous graviola leaf extract (AGE) (AGE 50: hyperlipidic diet + 50 mg/kg AGE. AGE 100: hyperlipidic diet + 100 mg/kg AGE. AGE 150: hyperlipidic diet + 150 mg/kg AGE) (depicted on the graph from the first day of treatment up to the 24th week). Values represent mean ± mean standard error. * *p* ≤ 0.05, ** *p* ≤ 0.01 and *** *p* ≤ 0.001 vs. SHAM SALINE. Student t-test (Figure 1A) and ANOVA followed by post Tukey test (Figure 1B).

However, with the HFD, the weight evolution evidenced a greater gain of body weight in the HFD group, with maintenance of a significant difference from the fourth week up to the 12th week (*p* = 0.005) compared to the control group (*p* ≤ 0.001) (Figure 1A).

Then, the animals of groups receiving a hyperlipidic diet began treatments with different doses of the extract or saline solution. At the 12th week, there was a significant difference in body weight in relation to the SHAM SALINE group (Table 3). However, this difference was not stable throughout the treatment. At the end of the 24th week, this group had a statistically similar body weight compared to the other groups (Figure 1B).

At the end of the treatment with the extract, the groups AGE 50 mg/kg and AGE 100 mg/kg presented a lower weight gain in comparison to the other groups (Table 3). The group AGE 50 mg/kg presented a statistical difference in relation to the SHAM SALINE group (*p* = 0.034). The group AGE 100 mg/kg had a significant body weight loss compared to the SHAM SALINE (*p* = 0.003) and HFD SALINE (*p* = 0.034) groups.

Food intake during the induction period was significantly higher in the SHAM group than in the HFD group (*p* ≤ 0.001). However, the caloric intake and the FEI were significantly higher in the HFD group than in the SHAM group (*p* ≤ 0.001) (Table 2). During the treatment period with the extract, similar results were observed for food intake, but daily caloric intake was significantly higher in the groups AGE 50 mg/kg (*p* = 0.007), AGE 100 mg/kg (*p* = 0.048) and AGE 150 mg/kg (*p* ≤ 0.001), compared to the SHAM SALINE group. However, the FEI was significantly lower in the group AGE 100 mg/kg when compared to the SHAM SALINE group (*p* = 0.021) and the HFD SALINE (*p* = 0.035) group (Table 3). That is, there was a lower feed conversion capacity into body mass in the group AGE 100 mg/kg.

### 3.4. Effects of AGE on Serum Biochemical Parameters

**Figure 2:**
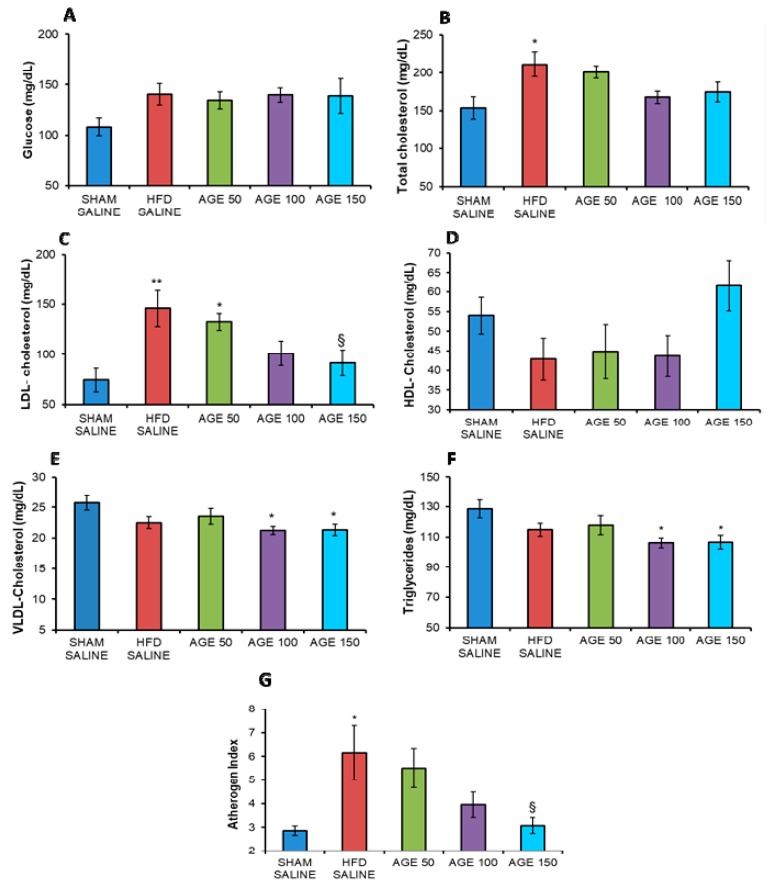
Evaluation of serum parameters. (**A**) Blood glucose (mg/dL), (**B**) total cholesterol (mg/dL), (**C**) LDL-cholesterol (mg/dL), (**D**) HDL-cholesterol (mg/dL), (**E**) VLDL-cholesterol (mg/dL), (**F**) triglycerides (mg/dL), and (**G**) atherogenic index of control animals (SHAM SALINE: standard diet + saline solution, HFD SALINE: hyperlipidic diet + saline solution), and of animals treated with aqueous graviola leaf extract (AGE) at 50, 100, and 150 mg/kg + hyperlipidic diet between the 13th and the 24th week of study. Values represent mean ± mean standard error. * *p* < 0.05, ** *p* < 0.01 vs. SHAM SALINE; ^§^ *p* < 0.05 vs. HFD SALINE. ANOVA followed by post Tukey test.

In this experimental model, the AGE was not able to decrease serum fasting glucose concentrations at the end of the study (*p* = 0.242) (Figure 2A).

The AGE was also not able to significantly change the total serum cholesterol and HDL concentration (Figure 2B,D). However, serum HDL-cholesterol levels showed a 30.35% increase in concentration in the group treated with AGE 150 mg/kg (61.57 ± 6.47 mg/dL), compared to the HFD SALINE group (42.88 ± 5.40 mg/dL) (Figure 2D). For total serum cholesterol, the percentage decrease in AGE-treated groups was 4.92% for AGE 50 mg/kg (200.84 ± 8.30 mg/dL), 20.54% for AGE 100 mg/kg (167.85 ± 8.22 mg/dL), and 17.49% for AGE 150 mg/kg (174.30 ± 13.10 mg/dL) compared to the HFD SALINE group (211.24 ± 16.33 mg/dL) (Figure 2B).

In this study, the decrease in LDL-cholesterol concentration in the AGE-treated groups seems to be directly associated with the dose given. That is, the higher the AGE dose, the greater the decrease. There was a significant difference (*p* = 0.038) between AGE 150 mg/kg in relation to the HFD SALINE group (Figure 2C). In addition, AGE was able to significantly decrease triglyceride concentrations in the treated groups at doses of 100 mg/kg (*p* = 0.026) and 150 mg/kg (*p* = 0.025) compared to the SHAM SALINE group (Figure 2F). There was also a decrease in VLDL cholesterol using AGE 100 mg/kg and 150 mg/kg (*p* = 0.030) compared to the SHAM SALINE group (Figure 2E). Regarding the atherogenic index, which evaluates the risk of developing cardiovascular diseases, the group AGE 150 mg/kg presented a significantly lower mean value (*p* = 0.025) than the HFD SALINE group. In addition, the HFD SALINE group presented a significantly higher value in relation to the SHAM SALINE group (Figure 2G).

### 3.5. Effects of AGE on Insulin Sensitivity and Glucose Tolerance

**Figure 3:**
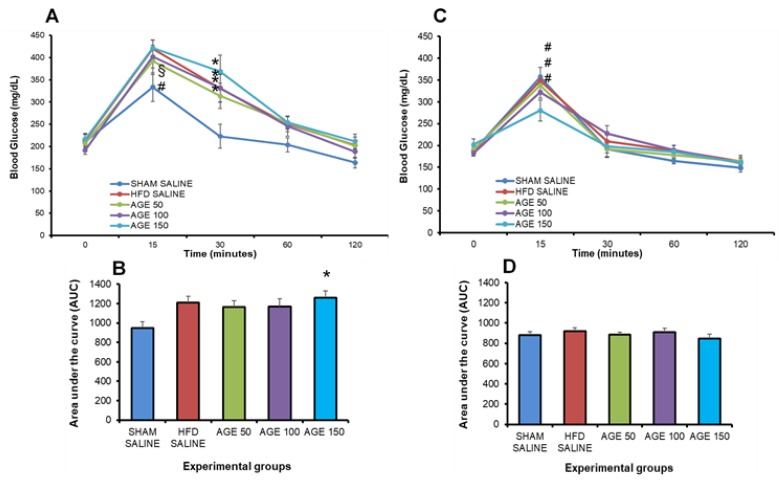
Evaluation of the glycemic profile before and at the end of the treatment with AGE. (**A**) Oral glucose tolerance test prior to the beginning of treatment (12th week). (**B**) Area under the curve (AUC) of blood glucose of animals evaluated prior to the beginning of treatment (12th week). (**C**) Oral glucose tolerance test at the end of treatment (24th week). (**D**) Area under the curve (AUC) of glycemia of animals evaluated at the end of treatment (24th week). SHAM SALINE: standard diet + saline solution. HFD SALINE: hyperlipidic diet + saline solution. AGE 50: hyperlipidic diet + 50 mg/kg of aqueous graviola leaf extract. AGE 100: hyperlipidic diet + 100 mg/kg of aqueous graviola leaf extract. AGE 150: hyperlipidic diet + 150 mg/kg of aqueous graviola leaf extract. Values represent mean ± mean standard error. * *p* < 0.05 vs. SHAM SALINE, ^§^ *p* < 0.05 vs. HFD SALINE, ^#^ *p* < 0.05 vs. AGE 150. ANOVA followed by post Tukey test.

The OGTT was performed prior to the beginning of the AGE treatment. No significant increases in fasting glycemia were observed between the hyperlipidic and the normolipidic diet groups. However, there was a significant increase (*p* ≤ 0.05) in the glycemia of animals at 15 min in the groups HFD SALINE and AGE 150 mg/kg, in relation to the group fed on a normolipidic diet (SHAM SALINE). At 30 min, all groups fed on a hyperlipidic diet had a significant increase in glycemia in relation to the SHAM SALINE group (Figure 3A).

The OGTT performed at the end of the experiment indicated that the AGE 150 mg/kg dose was able to significantly reduce blood glucose (*p* ≤ 0.05) at 15 min, in relation to the groups SHAM SALINE, HFD SALINE and AGE 50 mg/kg. However, there was no significant difference between AGE 100 mg/kg and AGE 150 mg/kg. The area under the curve did not indicate a significant difference for the comparison among groups (Figure 3B).

**Figure 4:**
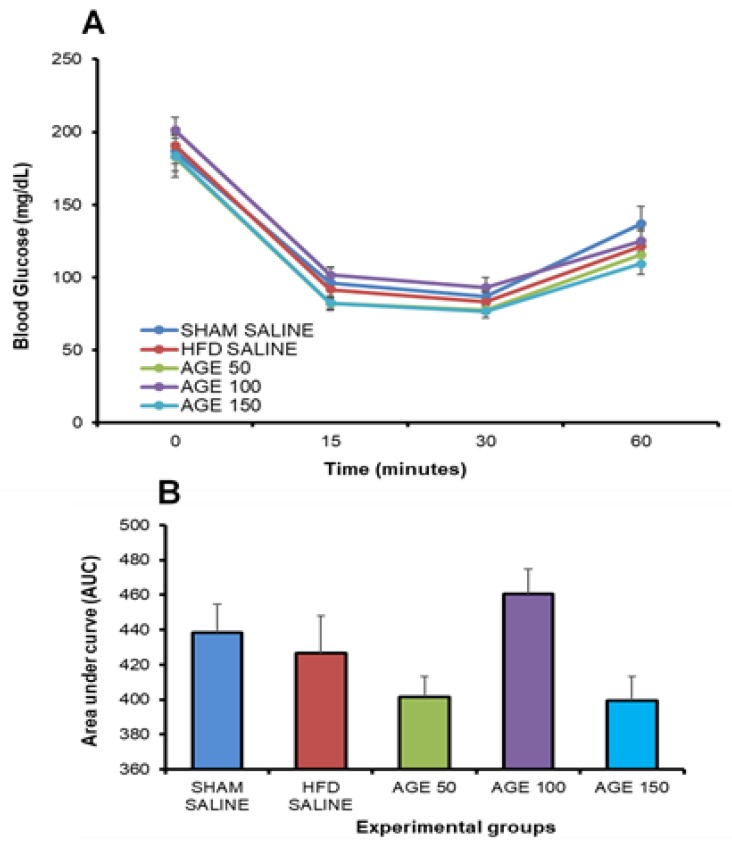
Evaluation of the glycemic profile at the end of the treatment with AGE. (**A**) Insulin sensitivity test performed at the end of treatment. (**B**) Area under the curve (AUC) of the insulin sensitivity test at the end of treatment. SHAM SALINE: standard diet + saline solution. HFD SALINE: hyperlipidic diet + saline solution. AGE 50: hyperlipidic diet + 50 mg/kg of aqueous graviola leaf extract. AGE 100: hyperlipidic diet + 100 mg/kg of aqueous graviola leaf extract. AGE 150: hyperlipidic diet + 150 mg/kg of aqueous graviola leaf extract. Each column represents the mean, and the bar represents the mean standard error. ANOVA.

The insulin sensitivity test performed at the end of the treatment with AGE or saline solution did not present a statistical difference in glycemia at the times analyzed after administration of insulin. This result is confirmed by observing the total area under the curve among the groups that received AGE or SALINE during the experimental period (Figure 4A,B).

### 3.6. Effects of AGE on Anti- and Pro-inflammatory Cytokines, Chemokines and Adiponectin

**Figure 5:**
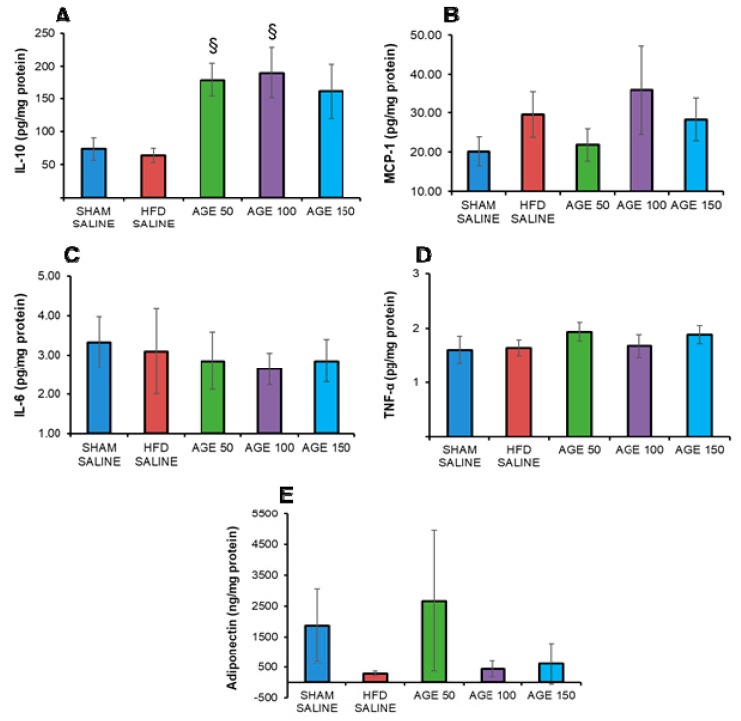
Effects of AGE on anti- and pro-inflammatory cytokines, chemokines and adiponectin. (**A**) Interleukin-10 (pg/mg protein). (**B**) Interleukin-6 (pg/mg protein). (**C**) Monocyte-1 chemotactic protein (pg/mg protein). (**D**) Tumor necrosis factor alpha (pg/mg protein). (**E**) Adiponectin (ng/mg protein) of control animals (SHAM SALINE: standard diet + saline solution. HFD SALINE: hyperlipidic diet + saline solution) and of animals treated with aqueous graviola leaf extract (AGE) at 50, 100 and 150 mg/kg + hyperlipidic diet between the 13th and the 24th week of study. The cytokines are measured in adipose tissue. Values represent mean ± mean standard error. ^§^ *p* < 0.05 vs. HFD SALINE. ANOVA/Tukey. Kruskal-Wallis test.

The animals treated with AGE showed an increase in IL-10 concentration, with a significant difference for the groups AGE 100 mg/kg (*p* = 0.021) and AGE 50 mg/kg (*p* = 0.042) when compared to the HFD SALINE group (Figure 5A). In analyzing MCP-1, no significant changes were observed between the groups studied (*p* = 0.840) (Figure 5B). As shown in Figure 5C,D, the levels of proinflammatory cytokines TNF-α (*p* = 0.640) and IL-6 (*p* = 0.768) also did not differ between study groups. Furthermore, there was no significant difference (*p* = 0.244) in the levels of adiponectin in the adipose tissue of mice (Figure 5D).

### 3.7. Effects of AGE on Fat Pads, Adiposity Index and Liver Weight

The AGE at the doses studied was not able to reduce the rate of adiposity of the animals. However, there was a decrease, although not significant, in the weight of fat pads of groups treated with aqueous graviola leaf extract: AGE 50 mg/kg: omental (22.22%), mesenteric (6.79%), retroperitoneal (8.79%), perirenal (9.41%); AGE 100 mg/kg: omental (11.11%), epididymal (14.34%), mesenteric (21.36%), retroperitoneal (31.04%); and AGE 150 mg/kg: omental (33.33%), epididymal (6.17%), mesenteric (6.08%), retroperitoneal (3.16%), and perirenal (2.35%), all compared to the HFD SALINE group (Table 4).

### 3.8. Effects of AGE on Liver, Pancreas, and Epididymal Adipose Tissue

**Figure 6:**
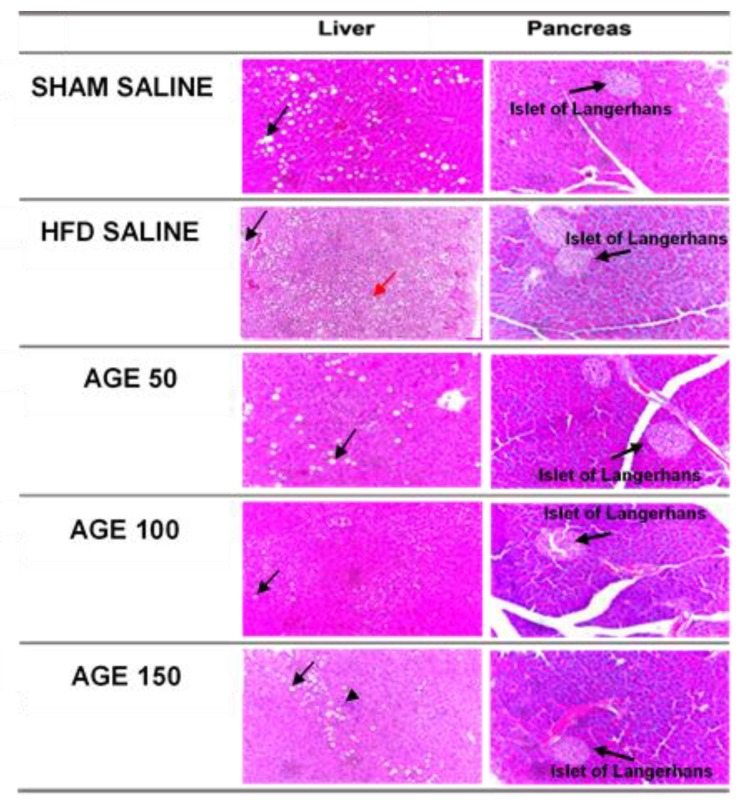
Histological analysis of the liver (Black arrows indicate hepatic steatosis, arrow head lobular inflammation and red arrows indicate ballooning) and pancreas of each experimental group. 20× magnification. Bar scale: 100 μm.

The histological analysis of the pancreas showed no statistical differences among groups regarding pancreatic acini (*p* = 0.400), Islet of Langerhans (*p* = 0.291), and inflammation (*p* = 0.458) (Table 5, Figure 6). However, the atrophy/necrosis was less frequent in the pancreas of animals treated with AGE, especially in the AGE 100 mg/kg group (Table 5).

Similarly, the liver histological analysis also showed that the treatment with AGE did not change the quantification of steatosis (*p* = 0.881), microvesicular steatosis (*p* = 0.501), lobular inflammation (*p* = 0.501), balloonization (*p* = 0.192), Mallory’s Hyaline (*p* = 0.408), apoptosis (*p* = 1.00), and glycogenate nucleus (*p* = 0.408) (Table 5, Figure 6). However, ballooning was more frequent in the HFD SALINE group compared to the groups that received AGE at different concentrations when fed on a hyperlipidic diet. Furthermore, hepatic steatosis was also frequent in the experimental groups that received a hyperlipidic diet or a normolipidic diet. However, the carbohydrate content in normolipidic diet was high, which may have contributed to this result (Table 6).

**Figure 7:**
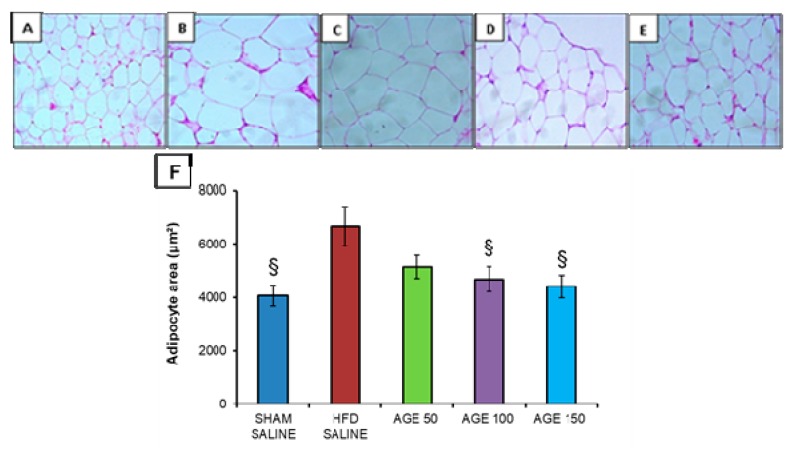
Histological analysis of the epididymal adipose tissue of each experimental group. (**A**) SHAM SALINE group. (**B**) HFD SALINE group. (**C**) AGE 50 mg/kg group. (**D**) AGE 100 mg/kg group. (**E**) AGE 150 mg/kg group. 20× magnification. Bar scale: 100 μm. **(F)** Adipocyte area (μm^2^) of the groups studied. Values represent mean ± mean standard error. ^§^ *p* < 0.05 vs. HFD SALINE. ANOVA followed by *post* Tukey test.

Regarding adipocytes, AGE at the dose of 100 mg/kg (4682.52 ± 476.91 μm^2^) and at the dose of 150 mg/kg (4410.54 ± 426.73 μm^2^) was able to significantly reduce the adipocyte area of the epididymal adipose tissue compared to the HFD SALINE group (6675.10 ± 736.87 μm^2^) (Figure 7).

## 4. Discussion

The choice of *Annona muricata* Linn for this study is because this plant is used for the treatment of obesity and its comorbidities. However, more scientific evidence is needed to support the notion that this plant extract can be used for treating obese patients [36].

Plants interact with the environment to survive and are influenced by many factors, such as pathogen attacks, temperature, circadian rhythm, water availability, nutrients, pollutants, and pesticides, all of which can cause stress. In response, plants produce secondary metabolites such as flavonoids, coumarins, saponins, alkaloids, tannins, and glucosinolates, among others. Thus, plants of the same species grown in different environments may present different concentrations of a certain secondary metabolic compound [37]. In a previous study, a high concentration of tannins and a medium concentration of flavonoids and saponins were identified in the methanolic and aqueous leaf extracts of *A. muricata* [38]. These substances were absent from the aqueous graviola leaf extract produced in this study. However, another study identified a low concentration of flavonoids and a high concentration of tannins, alkaloids, phenols, saponins, and phytosterols in AGE [39]. Thus, the different concentrations of the chemical composition of *Annona muricata* Linn leaves found in the literature and in our study may be related to the mentioned factors.

Studies have indicated that two hundred and twelve bioactive compounds have already been identified in *Annona muricata* Linn. Phenolic compounds are the major phytochemicals responsible for the antioxidant activity of *Annona muricata* Linn [36,40,41,42].

Acute toxicity tests using AGE found in the literature corroborate the results presented here. In the literature, the administration of a single dose of 2000 mg/kg and 5000 mg/kg of AGE to mice was not able to induce changes in animal behavior or mortality, or visible macroscopic changes in organs after euthanasia on the 14th day of the experiment [14].

Experimental models with modified diets can simulate pathophysiological changes in rodents that are similar to what occurs in humans. Such experiments allow understanding of the specific mechanisms of obesity and its metabolic changes. However, the feed composition and duration of experimental period have not been consistently established in the literature. In general, high-fat diets and physical inactivity are used in these models and are also the main risk factors for humans [40,43].

In experimental models with a high-fat diet, the increase in body weight is significant after two weeks of treatment, and after four weeks of induction this model shows different obesity phenotypes. However, long-term induction leads to obesity-related comorbidities such as moderate hyperglycemia and glucose intolerance [43]. Furthermore, in another model using C57BL/6J mice fed on a high-fat diet and 10% fructose after 16 weeks of treatment, the animals developed central obesity, dyslipidemia, arterial hypertension, insulin resistance, systemic oxidative stress, inflammation, and steatohepatitis. These are the main characteristics of metabolic syndrome [44].

In our study, we exposed mice to a 58% lipid diet for 12 weeks to induce obesity. After this period, our results indicated a significant increase in the weight of the HFD group compared to the SHAM group. The weight gain in the HFD group is consistent with the higher caloric intake evidenced by the FEI. Furthermore, at the end of the experimental period, we verified a significant increase in total and LDL cholesterol, and in the atherogenic index, of the HFD group in relation to the control group, which indicates that the model allowed the desired changes.

Among the various medicinal plants used for weight reduction, *Annona muricata* Linn is the second most used by the Brazilian population [16]. In our study, AGE at the doses 50 mg/kg and 100 mg/kg represented the popularly understood relationship between graviola and weight loss. Despite an observed decrease in body weight, no reduction in caloric intake was observed in the groups treated with AGE during the experimental period. Thus, weight reduction in this study is probably not related to a lower caloric intake.

Effective medicinal plants for weight loss have phenolic compounds among their chemical constituents, such as flavonoids, which modulate lipid metabolism and increase the rate of basal metabolism [45]. Quercetin and kaempferol stand out among flavonoids with an antiobesity effect. These were identified in AGE in the literature. We also found them in our study [42,46].

In the current study, regarding the cholesterol profile, no significant effects of the aqueous extract were observed on total cholesterol and HDL in two of the groups that received AGE. However, a significant increase in HDL cholesterol was observed in the group treated with AGE 150 mg/kg compared to the HFD SALINE group. We also observed a significant (*p* = 0.038) decrease in LDL-cholesterol, VLDL-cholesterol (*p* = 0.030), and triglyceride (*p* = 0.026) concentrations. In a study with streptozotocin-induced diabetic rats, AGE was able to significantly reduce plasma lipid concentrations. However, no difference was observed in relation to the diabetic group treated with 10 IU/kg of insulin [14].

The mechanisms of action of the aqueous extract of graviola on metabolism are not fully understood. However, several studies have reported isolated chemical compounds such as tannins, flavonoids, saponins, and coumarins, among other constituents, as being responsible for hypoglycemic, hypolipidemic, hypotensive, anti-inflammatory, and hepatic tissue changes, among other properties [47].

In our study, we observed a decrease in the atherogenic index after treatment with AGE, mainly in the group treated with 150 mg/kg. This decrease is directly related to the decrease in the development of cardiovascular diseases. This was associated with a decrease in triglycerides and LDL-cholesterol, and an increase in HDL-cholesterol, mainly in the 150 mg/kg group. Previous studies have shown that the antioxidant capacity of some substances can modify lipid metabolism and reduce inflammation, suggesting positive effects on cardiovascular diseases mainly by modulating oxidative stress. Furthermore, the high plasma level of the atherogenic index is related to small LDL-cholesterol particles. This is a predictor of conditions such as obesity, insulin resistance and inflammation, and consequently coronary artery disease, diabetes mellitus, and metabolic syndrome [48,49].

In our study, there were no significant effects of aqueous graviola leaf extract on capillary fasting glycemia evaluated in the oral glucose tolerance test performed at the end of treatment, and in the serum concentration of fasting glucose. When we calculated the area under the curve at the end of the experiment, we did not observe a significant difference in the comparison between the groups. However, there was a reduction in blood glucose levels at 15 min according to the oral glucose tolerance test in group 150 mg/kg. Some studies have demonstrated a significant decrease in plasma glucose concentrations after treatment with graviola extract in diabetic animals induced by streptozotocin or monohydrate aloxane [14,47,50,51].

Thus, the results found in our study do not indicate the effectiveness of aqueous graviola leaf extract on insulin resistance and diabetes mellitus type 2, in relation to the intake of a high calorie diet, high in saturated fat and simple carbohydrates and low in dietary fiber associated with sedentary lifestyle. However, further studies with AGE concentrations above 150 mg/kg may prove effective in reducing blood glucose, and therefore should be conducted.

Evidence shows that a greater fluctuation of glycemia induces endothelial dysfunction in diabetic or non-diabetic individuals, through oxidative stress resulting from an increase in free radicals [52,53].

Pro-inflammatory cytokines and chemokines, such as TNF-α, IL-6 and MCP-1, are required to initiate an inflammatory response. TNF-α is a cytokine that initiates the inflammatory response since it triggers the production of other cytokines, such as IL-6. On the other hand, anti-inflammatory cytokines, such as IL-10, are required to inhibit the synthesis of proinflammatory cytokines [54].

Previous studies have demonstrated that secondary metabolites present in plants, such as triterpenes, flavonoids and steroids, can modulate the inflammation and metabolic dysfunctions associated with obesity [55]. In our study, AGE did not change the levels of the inflammatory markers TNF-α, IL-6 and MCP-1 in adipose tissue. On the other hand, the AGE showed an anti-inflammatory effect due to a significant increase in IL-10 levels at the AGE doses of 50 and 100 mg/kg. In this study, the increased doses of AGE did not significantly interfere with TNF-α, IL-6 and MCP-1 levels. However, recent studies have demonstrated that IL-10 can exert anti-inflammatory effects via Janus kinase (JAK) signal transducer of activation 3 (JAK-STAT3), by binding IL-10 to the receptor on the target of the cell membrane—tyrosine kinase 2—leading to activation of the signal transducer and activator of transcription 3 (STAT3). However, further studies are needed to evaluate the possible effects of AGE on this pathway [56].

Adiponectin is a protein secreted by adipocytes. It exerts anti-diabetic, anti-atherogenic and anti-inflammatory effects directly. An increased expression may prevent and/or assist in the treatment of metabolic diseases related to obesity [57]. In our study, no significant effects of AGE were observed on adiponectin in adipose tissue. However, an increase of this protein was noticed in the group treated with AGE 50 mg/kg in relation to the other groups treated with AGE. Furthermore, the HFD SALINE group presented the lowest levels of adiponectin among the groups in our study.

Although the aqueous graviola leaf extract is able to induce a significant reduction in body weight according to the experimental model studied, and although there was a decrease in the weight percentage of all fat pads evaluated without significant differences in the comparison among groups, no decrease of visceral adiposity was observed at the end of the experiment when analyzing the weight of fat pads and the adiposity index. It is also possible to observe a significant decrease in the epididymal adipocyte area in the animals treated with AGE. Therefore, AGE attenuates the accumulation of lipids in mice, as was reported by another study after administration of blueberry and mulberry juice to C57BL/6 mice fed on a hyperlipidic diet for 12 weeks [58]. It should be noted that epididymal adipose tissue in mice is one of the major deposit areas of visceral fat [44].

In our experimental model, aqueous graviola leaf extract at the doses studied is not sufficient to prevent accumulation of liver fat and lesions to hepatocytes, as well as lesions to the pancreas. However, the ballooning of hepatocytes is less frequent in animals receiving treatment with the extract, as well as necrosis/atrophy of pancreatic acini. Thus, treatment with AGE is not able to avoid hepatic changes. However, it seems to protect the hepatocytes from morphological changes.

This may be related to a decrease in oxidative stress. In yet another study, the aqueous graviola leaf extract of *Annona muricata* Linn was able to protect pancreatic β-cells, and hence improve glucose metabolism, which was not visualized in our results [14].

## 5. Conclusions

In conclusion, no neurotoxic, behavioral, or mortality effects are produced by AGE in the acute toxicity test immediately after or during the post-treatment period. In addition, this study confirms the popular knowledge that graviola leaf tea reduces body weight and may also reduce cardiovascular risks, due to its beneficial effects in reducing plasma concentrations of LDL-cholesterol, VLDL-cholesterol, triglycerides, and the atherogenic index, while also attenuating the accumulation of body fat. In addition, in our experimental model, the results found do not indicate the effectiveness of aqueous graviola leaf extract on insulin resistance and diabetes mellitus type 2. However, the extract was effective in improving glucose tolerance in the higher concentration of the AGE. Furthermore, AGE has anti-inflammatory activity due to the increase in IL-10. However, it does not inhibit the expression of TNF-α, IL-6 and MCP-1. These data support the utility of conducting further studies aimed at identifying the active compounds of the aqueous extract of the aqueous graviola leaf extract, and at clarifying its mechanism of action.

## Figures and Tables

**Table 1:** Composition of experimental diets (g/kg diet).

Experimental Groups	AIN-93M Diet	High-Fat Diet (HFD)
Composition (g/kg)		
Cornstarch	620.69	320.69
Casein	140.00	140.00
Fat	-	320.00
Sucrose	100.00	100.00
Soybean oil	40.00	20.00
Fiber	50.00	50.00
Mineral mix	35.00	35.00
Vitamin mix	10.00	10.00
L-cystine	1.80	1.80
Choline bitartrate	2.50	2.50
Tert-butylhydroquinone	0.008	0.008
Energy (kcal/kg)	3802.8	5302.8
Carbohydrates (%)	75.81	31.73
Protein (%)	14.73	10.56
Lipids (%)	9.47	57.71
Calories/g of diet	3.80	5.30

**Table 2:** Initial and final weight, weight gain and food intake assessment during obesity induction between the first and the 12th week.

Parameter	Experimental Group	
	SHAM (*n* = 11)	HFD (*n* = 44)
Initial weight (g)	26.55 ± 0.55	26.52 ± 0.28
Final weight (g)	32.91 ± 0.99	37.87 ± 0.60 ***
Weight gain (g)	6.36 ± 0.95	11.34 ± 0.68 **
Food intake (g/day)	3.47 ± 0.05	2.73 ± 0.03 ***
Food intake (kcal/day)	13.18 ± 0.19	14.54 ± 0.16 ***
Feed efficiency index	0.0216 ± 0.0031	0.0501 ± 0.0033 ***

SHAM: Standard diet. HFD: Hyperlipidic diet. Values represent the mean ± mean standard error;, ** *p* ≤ 0.01, *** *p* ≤ 0.001 vs. SHAM SALINE. Student *t* test.

**Table 3:** Initial and final weight, weight gain, and food intake of control animals and animals treated with AGE between the 13th and the 24th week.

Parameter	Experimental Group				
	SHAM SALINE	HFD SALINE	AGE 50	AGE 100	AGE 150
Initial weight (g)	32.89 ± 0.94	38.09 ± 1.42 *	37.91 ± 1.09 *	37.82 ± 0.91 *	37.64 ± 1.43 *
Final weight (g)	35.91 ± 1.26	40.27 ± 1.42	37.82 ± 1.05	36.82 ± 1.33	38.27 ± 1.94
Weight gain (g)	3.09 ± 0.60	2.18 ± 0.26	−0.09 ± 0.53 *	−1.00 ± 1.14 **^,§^	0.64 ± 0.92
Food intake (g/day)	3.40 ± 0.43	2.62 ± 0.62 ***	2.76 ± 0.05 ***	2.70 ± 0.98 ***	2.83 ± 0.07 ***
Food intake (kcal/day)	12.89 ± 0.16	13.89 ± 0.33	14.65 ± 0.25 **	14.30 ± 0.52 *	14.99 ± 0.37 ***
Feed efficiency index	0.0109 ± 0.0021	0.010 ± 0.00128	−0.0006 ± 0.0023	−0.0036 ± 0.0050 *^,§^	0.0031 ± 0.0040

SHAM SALINE: standard diet + saline solution. HFD SALINE: hyperlipidic diet + saline solution. AGE 50: hyperlipidic diet + 50 mg/kg of aqueous graviola leaf extract. AGE 100: hyperlipidic diet + 100 mg/kg of aqueous graviola leaf extract. AGE 150: hyperlipidic diet + 150 mg/kg of aqueous graviola leaf extract. Values represent the mean ± mean standard error. In the same line, * *p* ≤ 0.05, ** *p* ≤ 0.01, *** *p* ≤ 0.001 vs. SHAM SALINE; ^§^ *p* ≤ 0.05 vs. HFD SALINE; ANOVA followed by post Tukey test.

**Table 4:** Effects of AGE on fat pads, adiposity index, and liver weight.

Parameter	Experimental Group				
	SHAM SALINE	HFD SALINE	AGE 50	AGE 100	AGE 150
Omental weight (g)	0.028 ± 0.009	0.018 ± 0.005	0.014 ± 0.004	0.016 ± 0.004	0.012 ± 0.004
Epididymal weight (g)	1.068 ± 0.127	1.506 ± 0.112	1.543 ± 0.783	1.290 ± 0.149	1.413 ± 0.169
Mesenteric weight (g)	0.496 ± 0.634	0.707 ± 0.092	0.659 ± 0.049	0.556 ± 0.122	0.664 ± 0.136
Retroperitoneal weight (g)	0.360 ± 0.053	0.728 ± 0.087 *	0.664 ± 0.053	0.502 ± 0.090	0.705 ± 0.125
Perirenal weight (g)	0.198 ± 0.039	0.255 ± 0.039	0.231 ± 0.026	0.275 ± 0.061	0.198 ± 0.036
Adiposity index (%)	6.080 ± 0.514	10.893 ± 0.481 ***	11.620 ± 0.387 ***	10.212 ± 0.797 ***	10.713 ± 0.779 ***
Liver (g)	1.225 ± 0.066	1.236 ± 0.056	1.213 ± 0.030	1.200 ± 0.058	1.185 ± 0.047

Values represent mean ± mean standard error. * *p* ≤ 0.05, *** *p* ≤ 0.001 vs. SHAM SALINE; ANOVA followed by post Tukey test.

**Table 5:** Results for changes observed in the pancreas of the animals in each experimental group.

Variable	Experimental Group				
	SHAM SALINE	HFD SALINE	AGE 50	AGE 100	AGE 150
Changes in the pancreas					
Islet of Langerhans (*p* = 0.291)					
No change	36.4 (4)	45.5 (5)	72.7 (8)	80.0 (8)	54.5 (6)
Discrete atrophy	9.1 (1)	0.0 (0)	0.0 (0)	0.0 (0)	9.1 (1)
Atrophy	18.2 (2)	36.4 (4)	9.1 (1)	0.0 (0)	0.0 (0)
Discrete hypertrophy	18.2 (2)	9.1 (1)	0.0 (0)	20.0 (2)	27.3 (3)
Hypertrophy	18.2 (2)	9.1 (1)	18.2 (2)	0.0 (0)	9.1 (1)
Pancreatic acini (*p* = 0.400)					
No change	81.8 (9)	72.7 (8)	90.9 (10)	100.0 (10)	90.9 (10)
Necrosis/Atrophy	18.2 (2)	27.3 (3)	9.1 (1)	0.0 (0)	9.1 (1)
Inflammatory cells (*p* = 0.458)					
No change	90.9 (10)	81.8 (9)	90.9 (10)	100.0 (10)	100.0 (11)
Insulitis	0.0 (0)	0.0 (0)	0.0 (0)	0.0 (0)	0.0 (0)
Perinsulitis	9.1 (1)	18.2 (2)	9.1 (1)	0.0 (0)	0.0 (0)

Data presented as relative frequency (absolute frequency). Value of *p* in the chi-square test.

**Table 6:** Results for changes observed in the liver of animals in each experimental group.

Variable	Experimental Group				
	SHAM SALINE	HFD SALINE	AGE 50	AGE 100	AGE 150
**Liver Changes**					
Steatosis (*p* = 0.881)					
< 5%	54.5 (5)	36.4 (4)	54.5 (6)	60.0 (6)	54.5 (6)
5 to 33%	36.4 (4)	36.4 (4)	36.4 (4)	10.0 (1)	36.4 (4)
34 to 66%	9.1 (1)	18.2 (2)	9.1 (1)	20.0 (2)	9.1 (1)
>66%	0.0 (0)	9.1 (1)	0.0 (0)	10.0 (1)	0.0 (0)
Microvesicular steatosis (*p* = 0.501)					
Absent	45.5 (5)	18.2 (2)	54.5 (6)	40.0 (4)	36.4 (4)
Present	54.5 (6)	81.8 (9)	45.5 (5)	60.0 (6)	63.6 (7)
Lobular inflammation (*p* = 0.919)					
Absent	63.6 (7)	72.7 (8)	81.8 (9)	70.7 (7)	72.7 (8)
<1 focus/field	36.4 (4)	27.3 (3)	18.2 (2)	30.0 (3)	27.3 (3)
2–4 focuses/field	0.0 (0)	0.0 (0)	0.0 (0)	0.0 (0)	0.0 (0)
> 4 focuses/field	0.0 (0)	0.0 (0)	0.0 (0)	0.0 (0)	0.0 (0)
Ballooning (*p* = 0.91)					
Absent	72.7 (8)	36.4 (4)	81.8 (9)	70.7 (7)	72.7 (8)
Few cells	27.3 (3)	63.6 (7)	18.2 (2)	30.0 (3)	27.3 (3)
Many cells	0.0 (0)	0.0 (0)	0.0 (0)	0.0 (0)	0.0 (0)
Mallory’s hyaline (*p* = 0.91)					
Absent	100.0 (11)	100.0 (11)	100.0 (11)	100.0 (10)	90.9 (10)
Present	0.0 (0)	0.0 (0)	0.0 (0)	0.0 (0)	9.1 (1)
Apoptosis					
Absent	100.0 (11)	100.0 (11)	100.0 (11)	100.0 (10)	100.0 (11)
Present	0.0 (0)	0.0 (0)	0.0 (0)	0.0 (0)	0.0 (0)
Glycogenate nucleus (*p* = 0.408)					
None/rare	100.0 (11)	100.0 (11)	100.0 (11)	100.0 (10)	90.9 (10)
Some	0.0 (0)	0.0 (0)	0.0 (0)	0.0 (0)	9.1 (1)

Data presented as relative frequency (absolute frequency). Value of *p* in the chi-square test.

## Supplementary Materials

The following are available online at https://www.mdpi.com/2072-6643/11/7/1509/s1, Figure S1: Body weight, weight of organs, food intake and water intake of animals in the control group and animals treated with AGE during the acute toxicity test.
