# Supplementary material for: Use of an Extract of Annona muricata Linn to Prevent High-Fat Diet Induced Metabolic Disorders in C57BL/6 Mice
Source: Nutrients. 2019 Jul 2;11(7):1509. doi: 10.3390/nu11071509 (PMC6682994; doi:10.3390/nu11071509)

Supplementary material

Acute oral toxicity

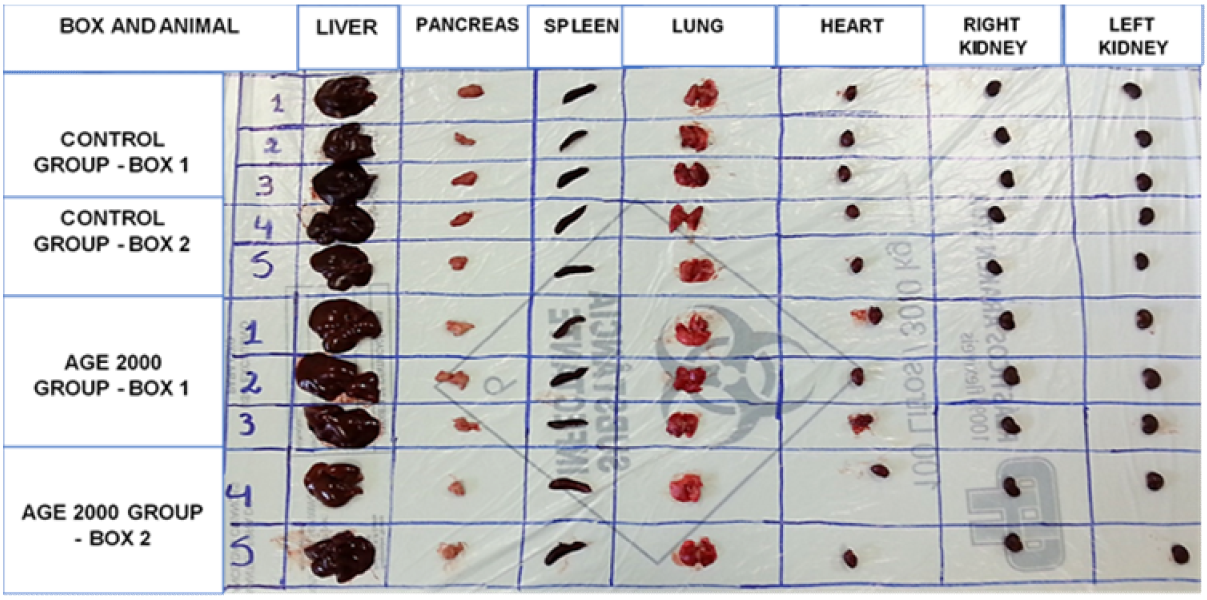

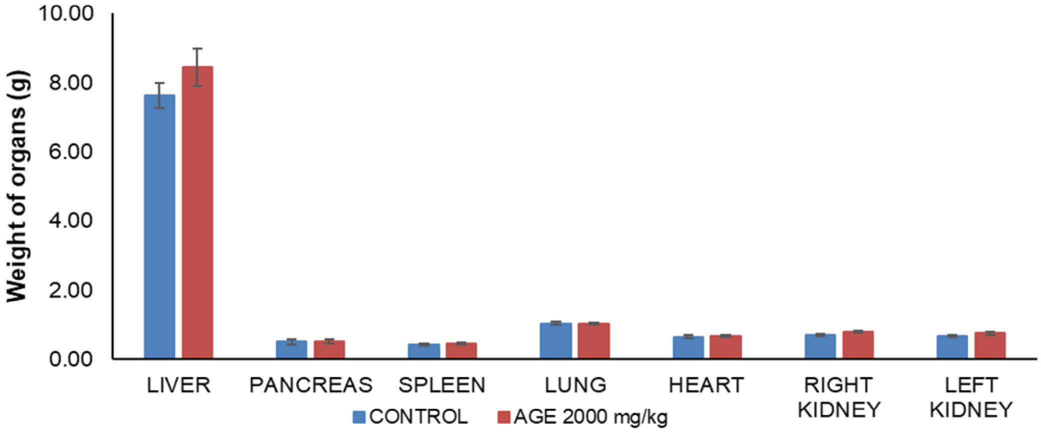

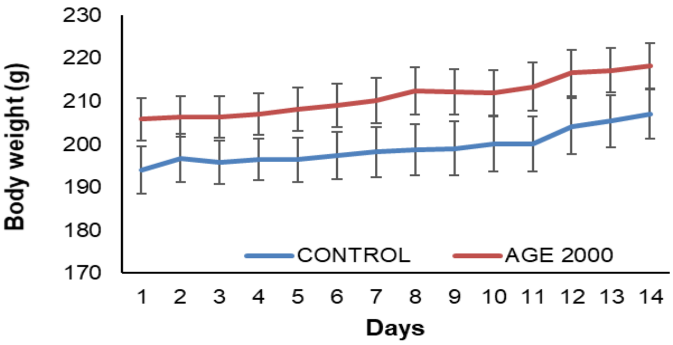

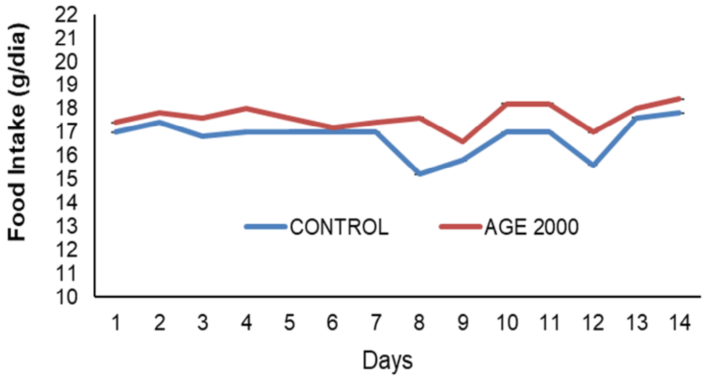

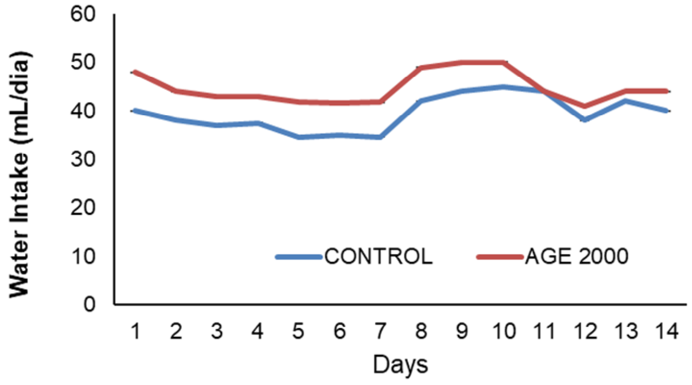

Supplement: Supplementary file 1 [file nutrients-11-01509-s001.pdf]
